# The potential role of cfDNA-related innate immune responses in postoperative bone loss after alveolar bone grafting

**DOI:** 10.3389/fimmu.2022.1068186

**Published:** 2023-01-04

**Authors:** Hanyao Huang, Renjie Yang, Bing Shi

**Affiliations:** ^1^ State Key Laboratory of Oral Diseases and National Clinical Research Center for Oral Diseases, Department of Oral and Maxillofacial Surgery, West China Hospital of Stomatology, Sichuan University, Chengdu, Sichuan, China; ^2^ State Key Laboratory of Oral Diseases and National Clinical Research Center for Oral Diseases, Eastern Clinic, West China Hospital of Stomatology, Sichuan University, Chengdu, Sichuan, China

**Keywords:** alveolar bone cleft, alveolar bone grafting, innate immune response, cell free DNA, TLR9, proinflammation

## Abstract

The purpose of treating alveolar bone cleft is to restore a normal maxilla structure. Multiple factors have been identified that can affect the success of alveolar bone grafting. However, with consistent treatment modifications, the surgical outcomes have been improved, but alveolar bone loss still exists. Thus, a new aspect should be found to solve this problem. As alveolar bone belongs to the periodontal tissues, the mechanism of the alveolar bone loss after bone grafting in patients with alveolar bone cleft may be similar to the development of alveolar bone loss in periodontitis. Cell-free DNA (cfDNA) has been demonstrated as a key promoter of alveolar bone loss during periodontal inflammation. We hypothesized that cfDNA-related innate immune responses could be a major inducement for postoperative bone loss after alveolar bone grafting. In this perspective, we preliminarily proved the potential association between cfDNA, TLR9 pathway, and alveolar bone grafting operation, and it might verify that surgical trauma could accumulate cfDNA, which can further activate cellular TLR9 signaling.

## Introduction

Patients with alveolar bone cleft need alveolar bone grafting to restore a normal maxilla structure, and the grafting of autogenous bone like iliac bone is still the most common choice (1, 2). However, bone loss after the surgery happens a lot (3). Clinical studies demonstrated that the operation age (1, 4), the structure of the alveolar cleft (5–7), and the pre- and post-operative maneuvers, especially poor management of oral hygiene (3), can affect the final outcomes of bone grafting. With consistent modifications of the treatment, the surgical outcomes have been improved, but the alveolar bone loss still exists (8, 9).

As alveolar bone belongs to the periodontal tissues, we hypothesize that the mechanism of the alveolar bone loss after bone grafting surgery might be similar to the development of alveolar bone loss in periodontitis. In periodontitis, tartar (mineralized plaque, soft scale, and food residue around the gingival sulcus) is the pathogenic factor that initiates the periodontal innate immune response and leads to inflammatory alveolar bone loss (10). For tartar, oral hygiene helps remove plaque and keep the tartar away (11), which will inhibit the innate immune response and stop the progress of inflammatory bone loss (12). Periodontal inflammation and related tissue destruction are more severer in patients with alveolar bone cleft than in those without alveolar bone cleft (13, 14), and the structure of alveolar defect can affect oral hygiene, then adversely exacerbate the periodontal status (15). Therefore, the local environment of the cleft is risky for enhancing bone loss. Oral hygiene, which can eliminate the local stimulus for periodontal inflammation, helps avoid bone grafting failure (16), which preliminarily supports our hypothesis that inhibition of local inflammation and innate immune responses could benefit bone grafting treatment.

Cell-free DNA (cfDNA)-related innate immune response is a key promoter to the progress of alveolar bone loss when periodontal inflammation happens (17, 18). Cell-free DNA (cfDNA) includes endogenous nuclear and mitochondrial DNA, and exogenous bacterial or viral DNA (19, 20). cfDNA plays the role of the ligands to DNA-sensing pathways, such as Toll-like Receptor 9 (TLR9), which can initiate the innate immune response, activate NF-κB signaling that leads to the secretion of proinflammatory cytokines like TNF-α, and cause inflammatory alveolar bone loss (17). In patients with periodontitis, cfDNA level in the gingival crevicular fluid is correlated with the degree of periodontitis (21–23). We recently confirmed that clearance of cfDNA can help alleviate alveolar bone loss by inhibiting TLR9 activation (17). As we have demonstrated the possible similarity between bone loss after bone grafting and the development of alveolar bone loss in periodontitis, herein, we hypothesize that cfDNA- and TLR9-related innate immune responses can also take part in the postoperative bone loss after alveolar bone grafting.

Postoperative bone loss after bone grafting possibly happens as the following: (1) surgery leads to sterile Inflammation, which increases the levels of damage-associated molecular patterns (DAMPs) (24, 25); (2) It is impossible to be a totally sterile environment in oral and maxillofacial surgery (26), which can lead to the increasing levels of pathogen-associated molecular patterns (PAMPs); (3) cfDNA levels will be increased because of the accumulation of DAMPs and PAMPs, and will consequently activate the TLR9/NF-κB pathway (19, 20) and may lead to the bone loss after alveolar bone grafting. In this perspective, we try to preliminarily demonstrate that cfDNA- and TLR9-related innate immune responses could happen after alveolar bone grafting in patients with alveolar bone cleft, which possibly is associated with postoperative bone loss, by showing pilot analyses of the pre- and post-operative levels of cfDNA in the gingival crevicular fluid (GCF) and serum of patients and use *in vitro* study to confirm that the cfDNA- and TLR9-related innate immune response can be activated after bone grafting surgery.

## cfDNA-related innate immune responses after alveolar bone grafting

### Increasing cfDNA levels after alveolar bone grafting in GCF and serum of patients

To determine whether cfDNA levels were increased in the body fluids of patients after alveolar bone grafting, 16 patients with alveolar bone cleft and without obvious periodontal inflammation were enrolled in this study. Patients were asked to have oral hygiene one month before the surgery, and the periodontal health of all patients with intact periodontium had no probing attachment loss, probing pocket depth ≤ 3mm, bleeding on probing < 10%, and no radiological bone loss (27). All the patients finished their cleft lip repair and palatoplasty before 2 years old. The surgery for the alveolar bone grafting was performed by the same surgeon, and the bone for grafting was collected from the iliac bone. Patient sample collection was performed with the approval of the Ethics Committee of West China Hospital of Stomatology, Sichuan University (WCHSIRB-CT-2020-272). All participants in this study signed an informed consent form before sample collection.

GCF and serum sampling were conducted before (preoperative) and 2 days after alveolar bone grafting (postoperative) (17, 18, 28). GCF sampling was performed on the teeth nearest to the cleft and surgical sites, which indicated the local inflammatory environment change at the surgical sites. The serum might demonstrate possible inflammatory environment change of the whole body because of the surgery, as surgery can cause damage to the tissue and lead to sterile inflammation. Extraction of cfDNA from GCF and serum was performed with a DNeasy Blood & Tissue Kit (QIAGEN, Germany). Concentrations of cfDNA in GCF and serum were measured with a Quant-iT PicoGreen double-stranded DNA Assay Kit. The statistical analyses were accomplished by Prism 8 (GraphPad). Paired t-test was used to compare the mean value between the two groups.

**Figure 1:**
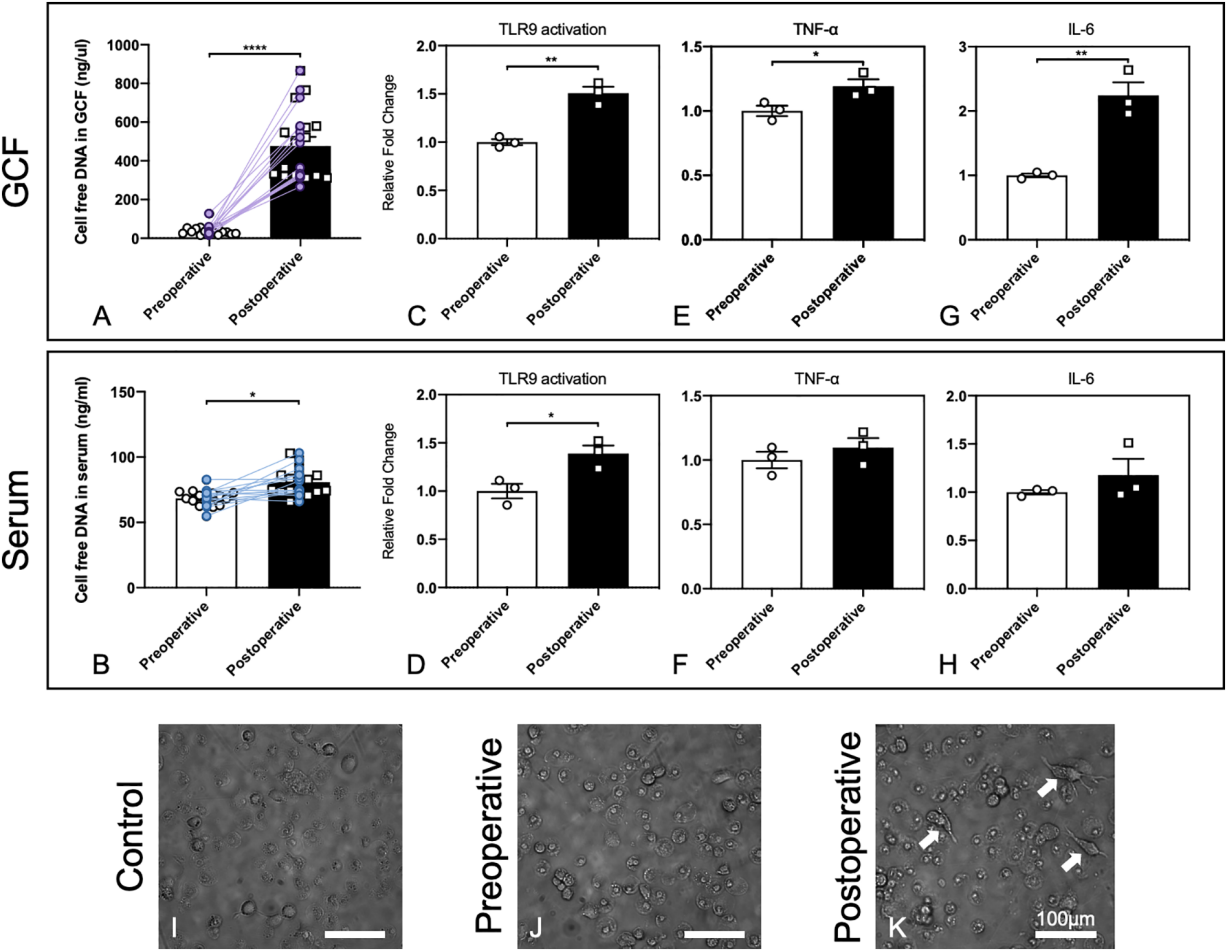
GCF and serum in patients after alveolar bone grafting induced stronger cfDNA-related innate immune responses. **(A, B)** Increasing cfDNA levels after alveolar bone grafting in GCF and serum of patients with alveolar bone cleft. Data are means ± SEM; *P < 0.05, ****P < 0.0001 assessed by paired t-test (n = 16). **(C, D)** Activation of HEK-TLR9 reporter cells by GCF and serum from patients with alveolar bone cleft before and after alveolar bone grafting. Data are means ± SEM; *P < 0.05, **P < 0.01 by Student’s *t-*test (*n* = 3). **(E, F)** TNF-α expression activated by GCF and serum from patients with alveolar bone cleft before and after alveolar bone grafting. Data are means ± SEM; *P < 0.05 by Student’s *t-*test (*n* = 3). **(G, H)** IL-6 expression activated by GCF and serum from patients with alveolar bone cleft before and after alveolar bone grafting. Data are means ± SEM; **P < 0.01 by Student’s *t-*test (*n* = 3). **(I–K)** Morphological changes of macrophages by patients’ GCF after alveolar bone grafting. Arrows show significant morphological changes in cells. Scale bar, 100 μm.

We found that 2 days after alveolar bone grafting, which involved the trauma to the periodontal tissues near the cleft and the trauma to the iliac bone, cfDNA levels in the GCF and serum increased significantly, while the increase was more significant in GCF (**Figures 1A, B**). Herein we confirmed that cfDNA could be associated with surgery, and the changing of cfDNA levels might be imputed to surgical damage to the local tissues and the potential infection in the oral environment. Based on the results, with cfDNA increasing in local sites of GCF, the following cfDNA-induced inflammation can happen, so then we carried out the *in vitro* study for preliminary exploration.

### Cellular TLR9 signaling activated by the body fluids of the patients after alveolar bone grafting

Next, we evaluated whether the GCF and serum could lead to higher activation of the cellular TLR9 signaling. Stable hTLR9-overexpressing HEK-Blue cells were purchased from *In vivo*Gen (San Diego, CA, U.S.A.) and were initially propagated in DMEM with 10% (v/v) FBS and maintained in growth medium supplemented with selective antibiotics (50 U/ml penicillin, 50 μg/ml streptomycin, 100 μg/ml Normocin, 2 mM L-glutamine). Before treatment, certain numbers of HEK-Blue hTLR9 cells (8 × 10^4^ cells/well hTLR9 cells) were seeded and cultured in basal DMEM overnight in 96-well plates, then stimulated with one microliter of human GCF and 20 μL of human serum from the patient who had alveolar bone grafting pre- and 2 days postoperatively, respectively. After 24 h, the activation of reporter cells was determined with the QUANTI-Blue medium with testing the secreted embryonic alkaline phosphatase (SEAP) activity. Student’s t-test was used to compare the mean value between the two groups. Different compositions and levels of cfDNA can lead to different levels of TLR9 response, so the higher cellular activation of HEK-Blue TLR9 cells means the cfDNA in the GCF and serum can stimulate higher TLR9 activation (17). Our results demonstrated that 2-days-postoperative GCF and serum induced significantly higher TLR9 activation in HEK-Blue TLR9 cells than the GCF and serum from the preoperative (**Figures 1C, D**), which verified that cfDNA from 2-days-postoperative GCF and serum could possibly cause a more significant proinflammatory response by inducing TLR9 pathway.

**Figure 2:**
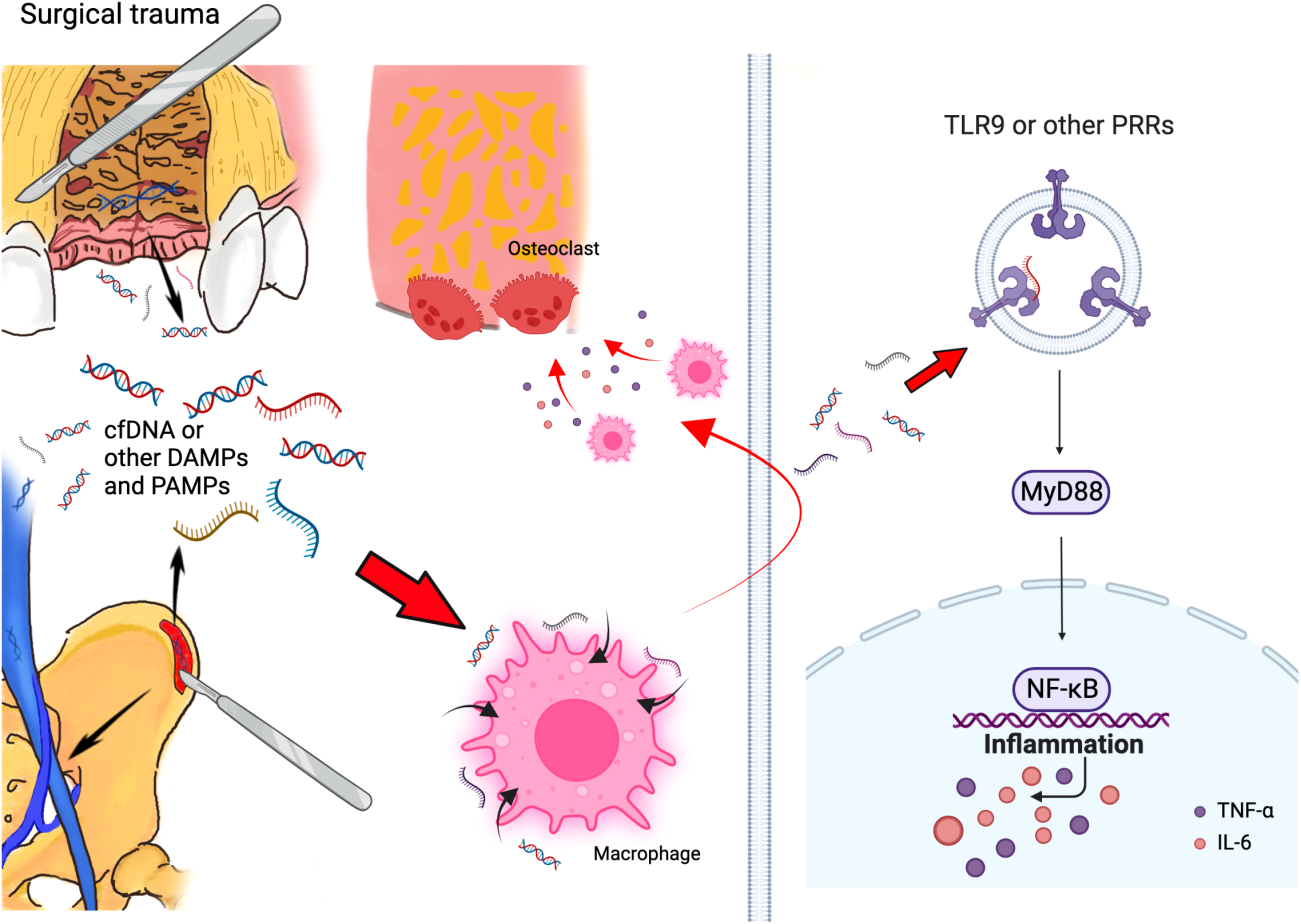
Schematic of mechanism that cfDNA and other DAMPs and PAMPs can promote bone loss initiated by surgical trauma during alveolar bone grafting. Surgery can accumulated cfDNA and other DAMPs and PAMPs, and these molecules can consistently activate the TLR9 and other PRR pathways, which activate the innate immune response and lead to bone loss after alveolar bone grafting. (Created with BioRender.com).

We then tested whether 2-days-postoperative GCF and serum caused a prominent increase in TNF-α and IL-6 levels in RAW 264.7 macrophages than the GCF and serum from the preoperative. RAW 264.7 cells were seeded and cultured in basal DMEM overnight at 2×10^4^ cells per well in a 96-well plate. One microliter of human GCF and 20 μL of human serum were then added into the well. After incubation for 24 h, the supernatants were collected and TNF-α and IL-6 levels were measured using ELISA kits purchased from Thermo Scientific (Waltham, Massachusetts, U.S.A). Paired t-test was used to compare the mean value between the two groups. The outcomes demonstrated that both TNF-α and IL-6 levels were increased, while the increase was significant by stimuli of GCF (**Figures 1E–H**). Together, these results suggested that cellular TLR9 signaling can be activated by the body fluids of the patients after alveolar bone grafting.

### Morphological changes of macrophages by the patients’ GCF after alveolar bone grafting

Macrophage polarization could be affected by the microenvironment in the periodontal tissues, and the phenotypes of macrophages could determine the final osteogenesis of the alveolar bone (29). Thus, we carried out a preliminary experiment by observing the morphological changes of macrophages by the stimuli of preoperative and postoperative GCF following the published protocol (17). Human monocyte THP-1 cells were purchased from ATCC (Manassas, VA, U.S.A.) and applied. THP-1 cells were cultured in RPMI-1640 media supplemented with 10% FBS and selective antibiotics. 8×10^4^ cells were plated in 96-well plates in 200 µL RPMI media plus 25 ng/mL phorbol myristate acetate (PMA) for 48 h, and one microliter of pre- and post-operative GCF was added during the final 18 h of treatment. After incubation, the morphology of cells was observed, which was altered by treatment with postoperative GCF, and dendrite-like change could be found (**Figures 1I–K**). This finding demonstrated postoperative GCF might have more stimulus that could alter the phenotypes of macrophages, but needed further investigation to confirm this hypothesis.

## Discussion and perspectives

Alveolar bone cleft is one of the most common craniofacial birth defects, often companied by cleft lip and palate (30). Alveolar bone cleft can influence the development of tooth and dental germ, including the quantity, morphology and position of tooth (31–33). Alveolar bone grafting is the standard treatment of clinics for alveolar bone cleft at present (34). A successful alveolar bone grafting has several purposes, including the bony continuity in the maxillary arch (5, 6), the stabilization of maxillary dental arch (7), the preservation for periodontal health of adjacent teeth (35, 36), the induction of permanent tooth eruption (1, 4) and implant placement (37). For getting successful operation outcomes, it’s indispensable to comprehend how multiple factors influence the surgical outcome.

Significant controversy for influence factors to a successful operation exists, in which the operation age (38), the cleft width (1, 39) and the cleft volume (40), presence of the lateral incisor, and the eruption and root development of the cleft-adjacent canine (41) are in a heated discussion. However, even though the aforementioned factors have been taken care of, the improvement in surgical outcomes was not significant yet, and postoperative alveolar bone loss still exists. Recently, poor oral hygiene became another hotspot in the success of alveolar cleft reconstruction surgery, which was similar to periodontitis (3, 16, 42). Thus, a new aspect based on this concept could be the potential for solving this problem.

As alveolar bone belongs to the periodontal tissues, we hypothesize that the mechanism of the alveolar bone loss in patients with alveolar bone cleft after bone grafting surgery may be similar to the development of alveolar bone loss in periodontitis. Another inflammation, peri-implantitis, which also happens in periodontal tissue, should also be mentioned in terms of our concept. It was found that a more pronounced inflammatory response was expressed in peri-implantitis than in periodontitis, which caused alveolar bone loss and failure of implant treatments (43, 44).

From our perspective, the progress of alveolar bone loss in surgical treatment for alveolar bone cleft can be similar to periodontitis and peri-implantitis. In periodontitis and peri-implantitis, during the innate immune response, the levels of PAMPs increase with dying bacteria (45); meanwhile, local inflammation causes cell death and accumulates DAMPs (46). Thus, in the inflammatory microenvironment of periodontitis and peri-implantitis, a collection of both PAMPs and DAMPs could continuously activate the immune systems and promote alveolar bone loss. In the situation of alveolar bone grafting in patients with alveolar bone cleft, surgeries in both the alveolar region and iliac bone region might contribute to the increase of DAMPs, accompanied by PAMPs generated in the oral cavity, which triggered the local immune response together and led to the postoperative alveolar bone loss (**Figure 2**).

Pattern-recognition receptors (PRRs), such as TLRs, which detect DAMPs and PAMPs, can initiate innate immune response (47, 48). Inappropriate activation of TLR9 happened in patients with periodontitis (49), as increased TLR9 levels can be found in their periodontal tissue (50). Meanwhile, TLR9-deficient mice are resistant to periodontitis (51, 52). We have confirmed that cfDNA can be a major source that enhances periodontal tissue destruction by activating TLR9 pathway, and targeting cfDNA and TLR9 pathway can help ameliorate periodontitis (17). Thus, based on the possible similarity between postoperative alveolar bone loss and periodontitis, we assumed that cfDNA- and TLR9-related innate immune responses could be a major inducement for postoperative bone loss after alveolar bone grafting. According to our outcomes, we preliminarily proved the potential association between cfDNA, TLR9 pathway, and alveolar bone grafting operation: Surgical trauma could accumulate cfDNA, and activate cellular TLR9 signaling *in vitro*.

Macrophages in the mononuclear phagocyte system are important in periodontal inflammation, as M1/M2 phenotypes can switch dynamically with the progression of periodontitis (53–55). We also observed morphological changes in macrophages with the stimuli of postoperative GCF, which is similar to the situation of periodontitis (17), which could be related to the M1/M2 phenotypes alteration. However, further study should be carried out to confirm this concept.

In this perspective, we hypothesize the potential enhancement by DNA sensing and TLR9-related innate immune responses to postoperative bone loss, and further experiments are necessary to elucidate the association between cfDNA, TLR9 pathway, and alveolar bone grafting operation associated with surgical trauma. Meanwhile, other PRR-related pathways should also be investigated in further study. For example, the TLR2 pathway has been confirmed both in the pathogenesis of periodontitis and peri-implantitis (56, 57); LPS and TLR4 pathway has been widely studied for the regulation in periodontitis and peri-implantitis (43, 56); and also other PRRs pathways should be considered (47, 48). Similarly, multiple cells, such as natural killer cells, mast cells, and neutrophils, should also be studied in the future as they are involved in innate immune responses (58). For the polarization of macrophages, further study should be concentrated on detecting phenotype markers by histology and flow cytometry. In summary, inflammation can be a potential source and target for managing postoperative bone loss after alveolar bone grafting.

## Concluding remarks

From this perspective, we propose that cfDNA can be the major source that enhances postoperative bone loss after alveolar bone grafting in patients with alveolar bone cleft, and targeting cfDNA and related pathways could be the potential therapeutic strategy to improve the treatment for patients with alveolar bone cleft.

## Data availability statement

The original contributions presented in the study are included in the article/supplementary material. Further inquiries can be directed to the corresponding author.

## Ethics statement

The studies involving human participants were reviewed and approved by The Ethics Committee of West China Hospital of Stomatology, Sichuan University (WCHSIRB-CT-2020-272). Written informed consent to participate in this study was provided by the participants’ legal guardian/next of kin.

## Author contributions

HH and RY contributed to the collection of data, analyses of the data, and writing and revising of the paper. HH and BS supervised the research. All authors contributed to the article and approved the submitted version.
